# When marital institutions break down: Impact and adaptation among the Enga of Papua New Guinea

**DOI:** 10.1017/ehs.2021.13

**Published:** 2021-03-01

**Authors:** Polly Wiessner, Nitze Pupu

**Affiliations:** 1 School of Human Evolution and Social Change, Tempe Arizona and Department of Anthropology, University of Utah, Salt Lake City, Utah, USA; 2 Enga Tradition and Tradition Centre, Wabag, Enga Province, Papua New Guinea

**Keywords:** Marital institutions, norm change, restorative justice, customary courts, Enga of Papua New Guinea

## Abstract

Institutions to regulate marriage and sexual mores are nearly universal across human societies to assure production and reproduction and weave the fabric of society. The stakeholders are many. What happens when marital traditions break down in times of rapid change? Taking a long-term perspective, we will first look at developments in marital institutions that occurred after the arrival of the sweet potato (ca. 400 BP) among the Enga of Papua New Guinea. Next, we will document changes in recent marital practices of 402 Enga women collected in 2007. With data from 270 public forums in customary courts applying restorative justice between 2008 and 2019, we will consider (a) the impact of the breakdown of marital institutions and (b) responses to adapt norms to new practices. In the absence of regulation by ‘traditional’ institutions, individuals pursue their own interests and passions with negative outcomes for families and communities. Communities, non-governmental organisations, churches and government throughout Papua New Guinea are seeking to adapt norms to new conditions. We consider both norm change resulting from community action via customary courts and what communities strive to preserve. Cultural institutions and accompanying norms are important factors in assuring production and reproduction; however, they can instill attitudes that inhibit adaptation.

## Graphical abstract

**Figure:**
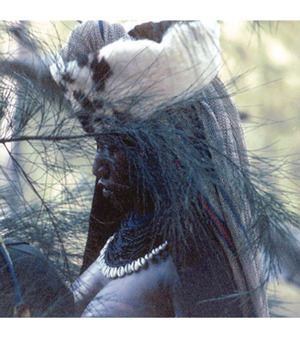

**Social media summary:** New technology, breakdown in marital practices and adaptive responses via restorative justice in Enga, Papua New Guinea.

‘Marriage is far too important to be left to the young’ (//Ukxa = Oma, //aru, Namibia, 2011)

## Introduction

Marriage is an almost universally found cultural institution with an extraordinarily wide variety of forms, in part because of the number of parties who have a stake in unions. As young people reach maturity, sexual attraction and love are potent forces in bringing couples together. Parents often have very different concerns. Following lifelong investment in children, parents seek in-laws who will treat their sons or daughters well and contribute to the family economy (Apostolou, 2007, 2017). Because marriage weaves the social fabric in most small-scale societies (Lévi-Strauss, 1969 [1949]), considerations of potential spouses are influenced by the location, resources and status of family into which offspring marry (Apostolou, 2007; Walker et al., 2011; Wiessner, 2009). Community members and affinal kin who contribute to ceremonies, dowry or bridewealth are concerned with social ties, economic prospects and the welfare of children who will assure the community's future. Complex politics often surround marriage arrangements, requiring young people to go through challenging rituals to qualify as suitable spouses. This is particularly true for young men, who may be kept out of the marriage market for years by older men in order to build cooperative, disciplined cohorts, reduce competition over leadership positions and free more women for polygynous marriages. In evolutionary studies, there has been a focus on mate preferences of young men and women in terms of physical attractiveness and other characteristics (Pillsworth, 2008; Sear & Marlowe, 2009; Borgerhof-Mulder, 2004); however, such preferences are often secondary given the many the stakeholders and agendas.

Throughout human history, marriage practices and accompanying values have been altered with shifts in economies, sex ratios, population pressure and political strategies, generating the great variation that has so intrigued anthropologists (Van den Berghe, 1990; Westermark 1891). In most cases, marital norms were gradually altered to fit changing circumstances brought on by such factors as altered sex ratios from the slave trade (Dalton & Leung, 2014), edicts against polygyny and cousin marriage by the Catholic Church (Henrich, 2020) or growing land shortage in the Himalayas (Rahimzadeh, 2020). Today, technology, transportation, money, education and other innovations have altered patterns of communication, interaction and marital options, often within a generation. Many youths pursue emotional needs through ‘love marriages’ (Shenk et al., 2016), seek unions that will promise better social and economic status or simply follow their passions in *de facto* relations. As the Rod Stewart song goes, ‘Young hearts be free tonight. Time is on your side. Don't let them put you down, don't let ‘em push you around, Don't let ‘em ever change your point of view.’ In societies with high educational standards and gender equality, both partners may benefit by breaking away from traditional norms and institutions. However, benefits depend on many factors: history, economy, ecology, gender relations, global integration and educational levels. In small-scale societies with more recent integration into the global economy, patriarchal norms, gender inequalities and low educational levels, the situation is different.

Our objectives are twofold. First, we will address the question of change in marital institutions in a small-scale society with a long-term case study among the Enga of Papua New Guinea. By marital institutions we mean the entire package of courtship gatherings, incest taboos and rituals to prepare men and women for marriage, as well as wedding formalities. Our study begins with the new marriage practices that were developed over the past 250–300 years to adapt to altered subsistence modes, large-scale migrations and conflicts, and to build the infrastructure for the growth of vast ceremonial exchange networks (Wiessner & Tumu, 1998). Next, we will turn to recent developments with the arrival of missions and the colonial administration starting around 1950 and the subsequent rapid changes from the early 2000s on related to mobile phones, improved transportation, guns and increasing involvement in the cash economy. These introductions radically altered patterns of communication and interaction, allowing a significant portion of young people to bypass traditional conventions. By 2007, bachelors’ ceremonies were no longer held and 34% of married women under 30 years of age in our study no longer engaged in traditional bridewealth exchanges to initiate lifelong affinal commitments. We will use data from 270 Enga customary court cases collected between 2008 and 2019 to measure the impacts of such changes and subsequent adaptations: how are old institutions adjusted to new practice and what do communities seek to maintain? Similar changes in marital practices, negative impacts and the quest for solutions are widespread throughout Papua New Guinea.

## Background: the Enga and their traditional marriage practices

The Enga of Papua New Guinea are a highland horticultural society who number about 500,000 today (Feil, 1984; Kyakas & Wiessner, 1992; Meggitt, 1965, 1977, 1991; Waddell, 1972; Wiessner & Tumu, 1998; Wohlt, 1978). They are master gardeners with their staple crop, the sweet potato, used to feed large human and pig populations. Approximately 90% of the population lives in rural areas. The Enga were/are embedded in two axes of kinship. The segmentary lineage system divides the Enga into tribes composed of exogamous patrilineal clans and sub-clans (Meggitt, 1965). Clans of 350–1000 are the units for most political action, including war. Marriage was largely clan exogamous and residence patrilocal. Paternal kin provide land, social identity, protection and lifelong support for many activities. Maternal and affinal kin furnish wealth from outside the clan and offer alternative residences and allies in warfare. Women become full members of their husbands’ clans and skillfully keep external ties alive. Enga women devote themselves primarily to childrearing, gardening, pig husbandry, and more recently, cash crops. Men shoulder the heavy work of making new gardens, fencing and house building; they also allocate much time to warfare, inter-clan politics and the ceremonial exchange of wealth. All male clan members are initially considered to be equals and go on to fiercely compete to attain the status of ‘big-man’ through ceremonial exchange, mediation, oration and organising events. Although women traditionally had influence in the private realm (Kyakas & Wiessner, 1992), male–female relations were characterised by male dominance. Today, both men and women engage in cash enterprises, as well as local and national politics and church activities.

Significant changes in marital practices occurred in Enga during pre-colonial history. After the arrival of the sweet potato released constraints on production some 350–400 years ago, there were substantial population shifts (Wiessner & Tumu, 1998). Groups formerly living from basic taro horticulture and hunting and gathering moved down into more fertile valleys, obtaining land on the basis of marriage ties. Myths and stories indicate that these were often troubled marriages owing to cultural differences, women from higher areas being portrayed as ‘wild’. With population shifts, major wars broke out, particularly in Central Enga, leading to large-scale migrations and extensive turmoil. Around the seventh generation before the present (Wiessner, 2002), private male rituals for growth were supplanted by new institutions: Sangai bachelors’ cults to bind fighting forces and control unruly youths. Displaced groups sought to put themselves back on the map of trade and exchange through the Great Ceremonial Wars after which marriages were forged to solidify alliances (Wiessner & Tumu, 1998). Neighbouring groups soon realised that bachelors’ cults standardised norms and values to facilitate marriage and exchange. The Sangai then spread rapidly throughout Enga. While there always seems to have been some form of bridewealth payments, these were elaborated hand-in-hand with exchange networks to build lifetime relations of mutual support.

The entire package of marital practices that had developed by two generations before contact with Europeans in central Enga, including courtship, bachelors’ cults and wedding formalities, is what we refer to as the ‘traditional’ baseline for change. At this time, boys went to live in men's houses at the tender age of 6–8 years. Around puberty, most young men attended a series of bachelors’ cults, Sangai, where they were taught mental and physical discipline and kept out of marriage until they had proven themselves to be responsible and mature adults between 25 and 30 years of age. Much emphasis was put on physical growth, purity and appearance, as well as skills in political thinking. During their years of periodically attending the Sangai, the bachelors were believed to be married to a spirit woman, represented by sacred objects, who would ensure that they grew into handsome, intelligent and mature men (Wiessner & Tumu, 1998). They were warned that the spirit woman would abandon them if they had pre-marital intimate relations with human women; should that happen, they would not be transformed. If one or more bachelors violated sexual norms, it was believed that the sacred objects representing the spirit woman would deteriorate so that the cohort of bachelors would have to jointly raise the wealth needed to purchase new ones from neighbouring clans. Contamination fears were strong: ‘My mother gave birth to five male children. Three of them married and two did not. They were told that if they married, they would become poor because a woman's vagina was the fire that burnt out the strength of a man (chuckle)’ (Ambone Mati, personal testimony, Enga Province, 1995). The spirit woman is the only example of ‘supernatural sanctioning’ beliefs in Enga culture (Norenzayan & Shariff, 2008), although today some branches of Charismatic Christianity in Enga are promoting the concept of punishment by the Christian god.

Through the Sangai, a lifelong sense of group loyalty was fostered within the cohort of young men. Although emphasis was put on relations between men and women, the power relations underlying bachelors’ cults were largely about older men controlling younger men and keeping them out of marriage until they were mature, disciplined clan members. Gender separation was also important for clan solidarity when men fought with the natal clans of their wives (Meggitt, 1977).

During the 3–7 years while young men attended bachelors’ cults, heavily chaperoned courtship parties and public dances (*sing-sings*) were the only events that allowed unrelated young men and women to get to know potential marriage partners (Kyakas & Wiessner, 1992). When bachelors were deemed marriageable, they conveyed their marriage preferences to their parents who, after approval, proceeded with the arrangements. Most young women married in their mid to late teens when they had developed an interest in several boyfriends during courtship parties, but had little further experience to guide their choices. Young women listened to their parents’ recommendations; if they strongly rejected a certain partner, parents conceded, knowing that the marriage would not succeed.

Once parents had agreed to a marriage, the two families met at a spot halfway between their respective clans. The groom brought pigs that his family had raised and formally asked the girl to marry him. The number of pigs should be sufficient but need not be too large (Kyakas & Wiessner, 1992). If the young woman refused the marriage, both groups went home. If she agreed, she returned to the groom's clan to engage in nightly parties for a month where she assertively sang songs asking the groom's kin to pledge contributions to her bridewealth. The bride's relatives generally accepted whatever they were given and distributed it to their kin to initiate ties between the two clans. A large bridewealth brought prestige to both parties; however, if the groom's clan had other important obligations at the time, a smaller bridewealth was acceptable. The purpose of marital wealth exchanges was not to ‘buy’ a wife (Eves, 2019), but to initiate lifelong commitments; the all-purpose currency of pigs was not storable so assistance from affines was called on throughout the lifecycle. In most cases, the bride's family gave some pigs in exchange. Bridewealth helped secure the marriage because upon divorce some amount had be repaid, unless the husband was severely abusive. A wedding feast sealed the marriage; during the next few weeks the groom went into seclusion to learn protective magic against the dangers of female contamination before the marriage could be consummated (Kyakas & Wiessner, 1992).

The bride became a full member of her husband's clan. Adultery was infrequent in the past, a fact that Meggitt (1991) attributes to men's fear of female contamination and women's fear of male rage. Men did beat their wives with sticks, but should a beating be too harsh, the wife would run home, leaving the husband with the pigs and children. He soon came with gifts to encourage her to return (Kyakas & Wiessner, 1992). Meggitt (1991, p. 102) found that, out of the several hundred women in his 1950–1955 studies in central Enga, there were only seven cases of domestic violence beyond simple beatings as men were heavily dependent on their wives for child care and domestic production. As Wohlt (1978, pp. 73–74) wrote, ‘I confess that after reading the literature on sexual antagonism for the New Guinea Highlands, I was somewhat surprised to find that quite a few Yumbisa marriages were characterised by genuine and persistent affection’.

Divorce could occur if the part or all bridewealth was repaid; however, divorce rates were low once the marriage was consummated (Kyakas & Wiessner, 1992), approximately 5% in Meggitt's studies (1991). Children belonged to the husband's clan, making women reluctant to divorce. Polygyny was permitted with the permission of the first wife for the 10–15% of successful big-men who sought to increase the productivity of their households and build political ties. Younger women rejected polygyny and had an arsenal of magic formulae to drive away second wives (Kyakas & Wiessner, 1992); however, more mature women often shared their husbands’ ambitions and raised the pigs for the bridewealth. Separate households were kept for each wife.

## Marriages practices in recent decades

Substantial changes were introduced by the Colonial Administration and missions in the 1950–1960s until independence from Australia in 1975. Formerly men and women lived in different houses. Men spent some family time in women's houses; women were forbidden to enter men's houses. Missions, largely Catholic, Lutheran or SDA, encouraged family houses and discouraged menstrual contamination beliefs and bachelors’ cults. As schools were opened, boys and girls from different clans had more interaction. In eastern Enga some bachelors sought more sexual freedom, attending bachelors’ cults but absenting themselves from rituals for the sacred objects representing marriage to a spirit woman (Schwab, 1995). Contamination beliefs remained influential in central Enga for two to three decades. Dr Paul Brennan, a linguist working in Enga, surveyed high school students in 1974 about deeds that men (*n* = 111) and women (*n* = 24) thought were bad or shameful. Seventy per cent of responses were related to being caught flirting, sexual inclinations or unclean sights such as seeing or being seen urinating, defecating or bathing naked. (Data given to the author by Paul Brennan in 2005.) When we began work in 1985, bachelors’ cults had been discontinued in the study area and such concerns were dying out. Churches supported Enga marriage traditions; the few who were married in the church also held traditional marriage ceremonies involving bridewealth exchanges. With increasing integration of the sexes, young people gained more say over whom they married, although parents still arranged marriages and ceremonies.

Starting in the mid-1990s, change accelerated. Fears of menstrual contamination further declined. With the breakdown of such belief systems, pre- and extra-marital sex became conceivable. However, it was developments in transport, communication and currencies that made changes in practice possible for many. Cash gives people many more options to pursue wants and needs than do pigs and traditional items. Stores provide foods that release heavy dependence on women's production for family sustenance. Affordable mobile phones became available in rural areas starting around 2007 and the constant circulation of people on public motor vehicles greatly alters patterns of interaction and communication between people of different ages, genders and social standings. With mobile phones, young people can arrange to meet up without parental consent. Configurations of power have been significantly altered by the adoption of guns in warfare, allowing youths to steer the course of wars (Wiessner, 2010; Wiessner & Pupu, 2012). Today large amounts of cash circulate from the sale of crops, employment, business, development programmes and the Porgera gold mine. Individualism is increasing: the traditional saying, ‘you need a person’, emphasising the value of every person, is being replaced by ‘money is life’. Videos portraying romantic exploits alter ideas about relations between the sexes. Downloads onto smartphones introduce the good, the bad and the ugly, including pornography and sorcery fears (Forsyth & Eves, 2015).

## Measures of change

In 2007 we interviewed 402 Enga women in central Enga who were married or sexually active in order to arrive at some measure of change. By then, many young people were choosing their own partners and co-habited without parental consent or formal marriage proceedings (Table 1). In the 51+ age group almost all women had been married traditionally with bridewealth, as had 62% of women between 31 and 50 years of age and 34% of those under 30. Of the 402 women interviewed, 16 (4%) said they did not want to marry but preferred to go around with different men (see Wardlow, 2006); another 18 (5%) were single mothers.

**Table 1.** Marriage with bridewealth and divorce by age group

*Age category*				
	Under 30	31–50	51+	Total
*Bridewealth*				
Number of women	138 (38%)	193 (52%)	37 (10%)	368 (100%)
Bridewealth paid	47 (34%)	120 (62%)	33 (89%)	198 (54%)
*Divorce*				
Number of women	130 (36%)	190 (53%)	38 (11%)	358 (100%)
Divorced	22 (17%)	45 (24%)	2 (5%)	69 (20%)

Data are missing on divorce for some women. Thirty-four women in the sample were single mothers or women who preferred to go with men but not marry.

Today, the increasing wealth inequalities generated by wage labour, business ventures and cash crops allow men who previously would not have had the resources for polygynous marriages to entice women as second wives, usually without paying bridewealth. Enga now consider couples who engage in repeated sexual relations and live together as ‘married’. There is much joking about the different kinds of modern marriage which captures the wide variety of ways that men and women ‘hook up’ (Wiessner, 2012).

- *Marriage bilong politics***:** a politician marries up to 20 women to get votes.
- *Chicken marriage*: a young man bribes parents with a chicken twice a week and has sex with the young woman while the parents are preparing and eating the chicken. For those with fewer resources, there are betelnut and Maggi soup marriages.
- *Road/market marriage*: the man seduces the woman with gifts on the road or in the market. After some time, he takes her away.
- *Car marriage*: the man tucks a K50 in his vest pocket with half of it showing and drives up in a vehicle. The woman gets in the front seat, ‘eats’ the K50 and drives away with the man. Later she finds out that he doesn't even own the car.
- *Video marriage*: young men and women watch videos and practice what they see.
- *Dina (credit) marriage*: a very common form of marriage where the man marries the woman on credit and has little incentive to pay bridewealth thereafter.
- *School marriage*: schoolmates decide to engage in sexual relations and then enter into a *de facto* marriage.

Bridewealth expresses recognition of the value of women in Enga and in neighbouring groups (Wardlow, 2006; Strathern, 1972). Most women who marry informally expect that bridewealth will eventually be paid; however, the incentive to pay bridewealth is diminished once couples are living together. Unpaid bridewealth is an ongoing source of marital tension. Some men do intend to pay bridewealth eventually, but others prefer uncommitted *de facto* relationships. Divorce rates were about 5% in Meggitt's studies conducted in the 1950s in the same study area (Meggitt, 1991). In our 2007 study, divorce rates were 5% for women in the 51+ years age group, 24% for women aged between 31 and 50 and 17% for women aged 30 and under (Table 1). Many divorces take place after 7–10 years of marriage when men engage in extra-marital affairs, claiming after the fact that they are marrying a second wife. Changes in sexual mores and marital practices are substantial. What are the repercussions? Here we will try get some measure the impact from 270 marriage disputes brought to customary courts in the Papua New Guinea justice system.

## The impact of change in marital institutions: complaints mediated in customary courts

The Papua New Guinea justice system is one of legal pluralism composed of formal national and district courts and customary courts (village courts) (Goddard, 2009; Gordon & Meggitt, 1985; Larcom, 2015; Narakobi, 1997; Pupu & Wiessner, 2018). The formal courts were imposed by colonial powers with written laws based on Western legal systems, procedures, courtrooms, strict rules of evidence, police, fines and prisons. They are not widely used outside of towns because most people want disputes to be mediated in open public forums and solutions to be based on restorative measures. Moreover, the requirements of formal courts for strict rules of evidence can rarely be met, witnesses are often afraid to testify and lawyers are expensive (Pupu & Wiessner, 2018). In 1984, the government sanctioned customary courts as part of the plural Papua New Guinea justice system. They were crafted from traditional dispute management based on *kastom* (custom) to mediate and correct minor wrongdoings such as marriage disputes, theft, property damage and assault, but not serious crimes such as murder, rape or arson. Village courts apply local ‘custom’ to settle disputes and achieve justice through compensation for harm done which satisfies communities**,** promotes harmony and restores the reputation and cooperation of wrongdoers. Custom was imprecisely defined and kept flexible to accommodate the many cultures of Papua New Guinea. There are two levels of customary courts in Enga, local village courts (VCs) and Operation Mekim Save (OMS), a branch of village courts unique to Enga established in the 1980s in each district to settle inter-clan disputes, control tribal warfare and keep the peace (Supplementary Material 1).

There are currently 153 VCs in Enga. Each serves one to four clans, 600–2000 people, who are usually from the same tribe; this grouping is what I will call ‘community’. Community members from different clans under one VC are densely linked through marriage ties and share church and government facilities. Village courts are presided over by panels of five or more magistrates, usually highly respected semi-literate male local leaders, and in the last decade, female leaders as well. Open air court hearings are held at central points in Enga villages two afternoons a week; Wabag OMS hearings are held outdoors in the provincial capital almost daily as they cover the entire district. Magistrates work tirelessly to keep the peace for very minimal salaries but they gain considerable status and respect for their work. Although most of the magistrates and communities in the study are either Catholic or Lutheran, magistrates are careful to separate custom from Christianity; in only about 4% of cases are Christian principles mentioned.

Village court hearings take place rain or shine, frequently interrupted by dogs, pigs or drunks. Opinions from both sides and third parties from community are heard. Between 30 and 200 people attend. The goal is to compensate the person who is wronged for suffering and loss, rather than punish the wrongdoer; of course, making up for harm done can be costly. Emphasis is placed on arriving at settlements that satisfy the community and restore cooperation. Customary court hearings are conducted in the Enga language, often employing complex symbolic speech. Summons are easily obtained by both men and women from the court clerk for a small fee. Men and women bring marital cases to VCs and OMS with similar frequency. For 132 OMS cases, 70 (53%) complaints were lodged by men and 62 (47%) by women; for 132 VC complaints, 61 (46%) were lodge by men and 71 (54%) by women. There are no set amounts for different infractions. Compensation awards are geared to context, levels of violence and ability to pay. Customary courts are used by people of all educational and economic levels from subsistence farmers to university graduates. The wealthy generally pay more.

Table 2 summarises the marital complaints brought to customary courts that reflect the often devastating impact of change. Most marital problems are first brought to local VCs because local community members have the knowledge to help solve them. Cases brought to OMS are those that risk sparking inter-clan conflicts, as well as complaints from couples living in towns and women who feel that they will not receive a fair settlement in their husbands’ clans. As is clear in Table 2, when rules regulating sexual mores and marriage can be bypassed, many men and women pursue individual passions and interests. For both courts, complaints concerning adultery backed by some evidence make up over a quarter of complaints; numbers of complaints accusing men and women are roughly the same, although for different reasons. Men engage in romantic adventures under the pretence of seeking second wives without the permission from their first wives. When caught, they must compensate the first wife for adultery; however, they can then go on to marry a second wife, sometimes abandoning the first. Such actions do not receive widespread approval, but there is little more that courts or community can do to prevent this. Prospective second wives often feel that they can drive away the first wife and have the husband to themselves.

**Table 2.** Complaints brought to village courts and the Wabag OMS customary court for intergroup conflicts

	Village courts	OMS
Male adultery	30 (14%)	25 (13%)
Female adultery	29 (14%)	29 (14%)
Domestic violence	49 (24%)	27 (14%)
Severe domestic violence	31 (15%)	21 (10%)
Alleged murder/responsible for death^a^	0 (0%)	10 (5%)
Lack of family support	17 (8%)	13 (6%)
Spouse departed	17 (8%)	27 (13%)
Broken betrothal/bridewealth	2 (1%)	10 (5%)
Defamation/threat	5 (2%)	7 (3%)
Property disputes	11 (5%)	22 (11%)
Second wives	14 (7%)	11 (6%)
Miscellaneous	4 (2%)	0 (0%)
Total	209 (100%)	202 (100%)

Some cases involved more than one complaint.

a Murder/death associated with domestic violence.

The reasons for female adultery are quite different, although women may also be assertive in pursuing their own goals (Mek et al., 2018). Some very young women marry rich older men in polygynous marriages, out of parental pressure or desire for an easier life, and then, disappointed, seduce younger men using their husband's money. When severely abused or betrayed, some women run back to their natal families and seek new partners out of sorrow or spite. Such cases can become very complex for the many factors that must be considered:

A young woman and a young man from a nearby clan started to live together but were not formally married. Their first child died. When the mother became pregnant a second time, she went to the Port Moresby hospital to deliver and died in childbirth, though the infant survived. The young woman's father took the husband to court to request compensation for the death of his daughter. The husband said the child was not his and refused liability on the grounds of adultery. As the case was heard, evidence came out that he never paid bridewealth, never paid compensation to maternal kin for the death of the first child (a serious breach of Enga ethics), and that he had beaten her several times using unreasonable force and destroyed her possessions. The case was adjourned three times to end with a settlement for the husband to pay 80 pigs and 20,000 PGK for the death of his partner which was attributed to ‘being driven by sorrow to her eventual death’. The magistrates upon handling down a settlement after several hearings said, ‘In Enga women were respected and are beginning to gain even more respect in their communities today. Enga are emotional people and they express mercy, love, and affection. None of these emotions were expressed by the husband.'

The sum was unusually high but both families were wealthy. OMS is permitted to mediate any amount of compensation, and the two clans were eager for a peaceful settlement, so there was no community protest (observed and described by Nitze Pupu in Wabag OMS, 2011).

Sexual freedom and adultery in turn set off a cascade of problems including high rates of domestic violence and the departure of wives. For VCs, 31 out of 80 (41%) cases of domestic violence for which we have information were set off by adultery accusations which were largely backed by evidence. Interestingly, the decline in norms regulating sexual freedom is having a roughly similar impact on unions regardless of the marriage type; rates of adultery are not significantly different in customary marriages than in *de facto* relationships (Table 3). This is in part attributable to the fact that some men who have paid bridewealth feel that they have the right to ‘seek second wives’ through adulterous relationships, bypassing their first wives. Other incidents of domestic violence were set off by disputes over property, particularly cash, work, insults, second wives and substance abuse (8%). Domestic violence can be life-threatening, particularly with substance abuse, as enraged husbands inflict injury with machetes or hot metal rods. Domestic violence cases brought to OMS were set off by a similar range of issues as those brought to the VCs, although often more complex ones. Incidents of domestic violence which were believed to have led to eventual deaths (5% of OMS complaints) were only brought to OMS, which have the authority to order higher compensation payments. Both VCs and OMS try to mediate the return of spouses who run back to their natal homes if their lives do not appear to be at risk.

**Table 3.** Domestic violence and adultery by marriage arrangement for village courts and OMS combined

	Domestic violence	Male Adultery	Female Adultery
Customary marriage	58 (47%)	27 (22%)	31 (25%)
*N* = VC = 55; OMS = 69^a^			
Total = 124			
*De facto* union, no children	15 (38%)	9 (23%)	7 (18%)
VC = 26; OMS = 14			
Total = 40			
*De facto* union with children	8 (22%)	8 (22%)	6 (17%)
VC = 14; OMS = 22			
Total = 36			
Polygamy	8 (42%)	2 (11%)	4 (21%)
VC = 11; OMS = 8			
Total = 19			
Total	89 (41%)	46 (21%)	48 (22%)
VC = 106; OMS = 113			
Total = 219			

a Number of cases for which we have information.

VC, Village court; OMS, Operation Mekim Save (higher customary court).

## Settling marital problems

The aims of the VC and OMS settlements for marital disputes via restorative justice are: (a) to try to stabilise the marriage in the interest of the couple, children and affinal relatives; and (b) to maintain peace and cooperation within and between communities. Because troubled *de facto* unions can ignite as many problems as formal marriages, they are now handled as marriages in both VCs and OMS to adapt to changing practice (Table 4). *De facto* unions, like formal marriage settlements, are frequently settled by compensation. Formal marriage cases and *de facto* unions without children are more frequently taken to OMS than are *de facto* unions because of issues around bridewealth return and child custody. VCs and OMS handle marital complaints somewhat differently. In the VCs, magistrates know about the conditions of the marriage, evidence is plentiful and magistrates consider the history of the relationship. As leaders chosen by communities, they must be must be careful to calculate settlements to make up for damage done rather than to punish one party, else they risk being accused of bias. In OMS, where magistrates have less information and marriage disputes can lead to inter-group conflicts, magistrates either mediate compensation agreements or adjourn the case several times until the two parties decide to settle by themselves. Neither VCs nor OMS compensation settlements ensure that the conflict is resolved, but simply give the relationship another chance, make up for harm done and assuage affines, thereby reducing risks of retaliation.

**Table 4.** Settlement by marriage arrangement for village courts and OMS combined

	Compensation	Divorce	Settle alone^b^	Adjourned	Total
Customary marriage	67 (55%)	20 (16%)	16 (13%)	19 (15%)	122 (100%)
*N* = VC = 55; OMS = 67^a^					
*De facto* union, no children	17 (43%)	15 (38%)	6 (15%)	2 (5%)	40 (100%)
*N* = VC = 26; OMS = 14					
*De facto* union with children	19 (54%)	7 (20%)	3 (9%)	6 (17%)	35 (100%)
*N* = VC = 13; OMS = 22					
Polygamy	7 (39%)	7 (39%)	4 (22%)	0 (0%)	18 (100%)
*N* = VC = 11; OMS = 7					
Total	110 (51%)	49 (23%)	29 (13%)	27 (13%)	215 (100%)
*N* = VC = 105; OMS = 110					
Total = 215					

a Number of cases for which we have information for village courts and OMS. Some cases are pending.

b Settle alone: after hearing, parties decide to go home and settle by themselves.

One VC case and three OMS cases were dismissed for no evidence and not included in the above tallies.

Compensation payments for adultery are usually near the VC limit, PKG 1000 (ca. US$300), but if they are accompanied by severe domestic violence several pigs may be added. Compensation for domestic violence is geared to both what the defendant can pay and to the severity of the injury inflicted. Despite the high frequency of compensation settlements, divorce is granted in 23% of hearings, usually only after marital conflicts have been brought to court several times, after life-threatening domestic violence or when both parties wish to divorce. *De facto* marriages with no children are dissolved at higher rates than customary marriages (*χ*^2^ = 6.43, *p* = 0.011) and more easily; most customary marriages require some bridewealth repayment.

Community members participate actively during customary court cases, serving as witnesses, giving opinions and making suggestions (Wiessner, 2020). The following case gives an interesting example of how the community pitches in to try to rescue unions.

The couple were involved in a *de facto* relationship for three years. The defendant was a marijuana smoker and drinker of homebrew. He would return late at night and sometimes beat his partner to the point of unbearable pain. She summoned him to the VC. A community member who was a teacher told him not to smoke marijuana because everyone in the community knew he was an addict. A teacher also told him not to drink homebrew, warning that such behaviour, if not controlled, would eventually erode their marriage and destroy his sanity. The teacher said that his own father also died when he was young and didn't leave him any inheritance, so he had to make it on his own through self-discipline and learning from interactions of people in the community.

In the presence of VC magistrates and community leaders, the dispute was discussed and people in the crowd felt concerned for the young couple. Immediately contributions poured in for a total amount of PGK 685. The amount was collected and offered to the complainant by the chairman who said it was the function of the court to ensure that marriages were maintained and protected. It was not proper or even unlawful to dissolve a marriage unless the union was not possible under current conditions. Then he issued an order to the defendant to stop substance and wife abuse. The father of the complainant expressed satisfaction over the settlement consenting that his daughter remained with the defendant as her rightful husband. One contributor said that no bridewealth had been paid in this union, something that was a bit of an embarrassment to the community. He thought the settlement involving community contributions (to help make up for unpaid bridewealth) was appropriate to support the marriage and maintain unity in the community. (Collected in Wakumale Village Court by Nitze Pupu, 2017.)

For 78/132 of VC marriage cases (59%) and 83/129 OMS cases (64%), both parties were satisfied and felt the dispute was settled for the time being. Once people have aired their complaints in hearings, they may be ready to work out marital problems themselves. However, what happens when there is dissatisfaction? Cases can be appealed but rarely are. Amounts of compensation awarded are not fixed nor are they followed up for non-compliance unless there is a subsequent complaint (less than 5% of cases). There are limits on settlement amounts that can be awarded in the VC and community members often feel that compensation orders are too low. Members then step forward to assist with raising a higher payment to make sure that the injured party and kin are satisfied. To date we have followed up on compensation paid for 23 marriage settlements from VC cases, asking who contributed to the compensation, how much they gave and why. It was only possible to get the above information for major contributors; small amounts are given casually.

The actual amounts of compensation paid for all types of infractions are often considerably more than ordered (Wiessner, 2020) with the goal of ‘making the complainant happy’ (Table 5). Amounts that exceeded the settlement order were given for both formal customary marriages and *de facto* marriages. For the five cases of underage sex and pregnancy, community members stepped forward to top up the compensation order, save reputations, prevent retaliation and support youths to continue in school. In small-scale societies where people obtain most of their resources from collective activities, sharing and exchange, every person is of value (Gurven, 2001; Hill & Kaplan, 1999). Compensation paid in customary courts is embedded in the much larger sphere of bridewealth exchanges, child growth payments, war reparations and funeral exchanges. Receiving assistance does not absolve wrongdoers of responsibility; those who receive assistance are aware that they must help out when the donors are in need.

**Table 5.** Follow-up of marriage settlements by marriage arrangement for Village Courts^b^

	More than ordered			Less that ordered		
	*n*	Mean	Range	*n*	Mean	Range
Married	5	K2194	K70–6600	1	K300	0
*De facto*/no children	5	K1969	K145–7500	2	K1010	K900–1120
*De facto*/children	2	K1135	K310–1960	0	0	0
Polygamy^a^	1	K529	K218–840	0	0	0
Brawl	3	K4200	K3400–5000	3	K493	K200–900
Total	16	K2033	K70–7500	6	K633	K200–1120

a The exact amount ordered was paid in one polygamous marriage, K1000 to compensate the first wife for marrying a second wife.

^b^For OMS cases we have only followed up murder and other serious crimes for which entire clans contribute and settlements run up to more than 100 pigs and PKG 100,000.

**Table 6.** Longer-term outcome for 90 marital disputes brought to village courts between 2008 and 2019

	*N*	Still married	Divorced^a^
Customary marriage	47	17 (36%)	30 (64%)
*De facto* union/no children	23	3 (15%)	20 (87%)
*De facto* union with children	12	4 (33%)	8 (67%)
Polygynous marriage	8	4 (50%)	4 (50%)
Total	90	28 (31%)	62 (69%)

a Most divorces are only granted after numerous complaints heard indicating recurring problems, particularly domestic violence.

**Table 7.** Remarriage after divorce by marriage arrangement

Remarried					
	Man only	Woman only	Both	Neither	Total
	*n*	*n*	*n*	*n*	
Customary marriage	2 (6%)	8 (27%)	18 (60%)	2(7%)	30 (100%)
*De facto* union, no children	2 (11%)	1 (5%)	15 (79%)	1 (5%)	19 (100%)
*De facto* union with children	0 (0%)	3 (37%)	5 (63%)	0 (0%)	8 (100%)
Polygynous marriage	0 (0%)	0 (0%)	3 (75%)	1 (25%)	4 (100%)
Total	4 (7%)	12 (20%)	41 (67%)	4 (6%)	61 (100%)

The above data come from court cases conducted between 2008 and 2019. The follow-up was conducted in January 2021.

In six cases less than the order was paid, even though settlement orders are reduced for poorer families. The reasons for failure to raise the compensation include poverty, failure to help others and above all, frequent troublemaking. In this way, repeated offenders or non-cooperators are marginalised, although not given any direct punitive sanctions. Those who frequently make trouble but are otherwise generous and cooperative community members continue to be supported. When wrongdoers cannot raise the full amount, community members pressure the complainants to accept a lower payment, praising them for restoring community harmony. For polygynous marriages, fewer are willing to contribute as polygynists are supposed to have sufficient funds to support multiple wives. The category ‘brawl’ involves marriage disputes that expanded to include third parties who were seriously injured, thus the high payments. Lower payments for brawls were those between co-wives.

Who contributes and why? Of the 68 contributors interviewed, 25% were immediate family, 38% aunts, uncles and cousins, 9% affines and 28% clan members from other sub-clans and other more distant kin. Although most help was given by close kin, approximately a third of assistance was given by affines and more distant supporters in the clan or neighbouring ones to help solve community problems. Primary reasons given for helping (*n* = 68) include: to solve family problems (32%), to restore peace and harmony to the community (32%), because the wrongdoer had given help to the contributor in the past (18%), to exercise leadership by problem solving (15%) and to prevent arrest for a serious offence (3%).

Although *de facto* unions initially anger parents and dismay community members, if they persist, people find ways to include the couple in traditional exchanges through assisting when they are in trouble. Such help is usually reciprocated in some way at a later date when the donor must raise payments for bridewealth, war reparations or funeral exchanges. By supporting unions in compensation settlements, *de facto* relationships may be stabilised and drawn into the broader stream of give and take. Up to 70% of cash in Enga circulates widely for many different kinds of exchanges, including compensation, and never reaches the coffers of stores. These exchanges weave the fabric of Enga society and secure families.

## Outcomes

What happens to troubled unions in the long run? How successful are VCs? One member of our team, Larsen Kyalae, has so far followed up 90 of the 132 disputes between marriage partners in our VC sample collected between 2008 and 2019. Follow-up of OMS cases is still a work in progress, hindered by conflicts in the area that make travel beyond local communities dangerous. Of the 90 unions, 28 (31%) couples were still married and 62 (69%) had divorced (Table 6). The VCs are thus more successful in dissolving tumultuous marriages to avoid further harm than in saving them. *De facto* marriages with no children had significantly higher divorce rates than customary marriages or *de facto* marriages with children (*χ*^2^ = 4.04, *p* = 0.044). Eighty-four per cent of women and 89% of men who were divorced in *de facto* marriages with no children were remarried by 2021 (Table 7). Such unions are easily formed and dissolved, although usually not without ample emotional hurt, violence and disruption to community. Most divorced men and women from customary marriages, *de facto* marriages with children and polygynous marriages also remarried – 67% of men and 88% of women. Divorce and remarriage, stigmatised in the past, are widely accepted today. Remarriage does not usually involve more than minimal bridewealth exchanges.

## Discussion

Marital institutions among the Enga have been regularly adapted to changing circumstances over generations. After the arrival of the sweet potato, change came at the institutional level to adjust to population shifts, a growing surplus economy and expanding ceremonial exchange networks. Elders devised bachelors’ cults to discipline young men and gain strict control over sexual mores and marriage. Elaborate bridewealth exchanges were devised to provide the backbone for rapidly expanding ceremonial exchange networks involving tens of thousands of people and pigs (Wiessner & Tumu, 1998).

After the missions and the colonial administration were established in the 1950s and 1960s, bachelors’ cults and residential arrangements separating the sexes were dismantled within a span of two to three decades in central Enga. Recently, as patterns of communication and interaction are altered by new technology, the majority of couples have ignored former sexual mores and bypassed traditional marital institutions involving bridewealth payments. The impact of these changes was and is, in many cases, devastating. With fewer restrictions, both men and women pursue immediate sexual desires; as a result, brutal domestic violence, adultery, and high divorce rates are commonplace, disrupting families and community harmony. Those most negatively affected are women and children, who may be abandoned for new partners. Some are saddled with the heavy economic burden of child rearing in a rural economy where there is no official child support. The recent processes of change differ from those of pre-colonial times. While formerly the development of institutions by leaders drove new marriage practices, in recent times technology and outside influences have allowed new practices to drive institutional change.

What has changed and what has remained the same? What do Enga seek to maintain? The strict beliefs regarding female menstrual contamination have evaporated except for some practices regarding food preparation. Sexual relations before marriage, although discouraged, are becoming common practice; adultery is not condoned but is frequent. The current acceptance of *de facto* unions and ease of divorce and remarriage, which were stigmatised in the past, suggest a true norm change to acceptance of pre-marital cohabitation or ‘trial marriages’, as is common in many parts of the world (Howell, 2000; Du & Mace, 2019).

The number of marriages with bridewealth exchanges has declined significantly but they still remain the ideal for the respect shown to women and ties built between families (Henry & Vávrová, 2020; Wardlow, 2006). In *de facto* relationships women and their kin hope that bridewealth will eventually be paid; customary courts cannot order such voluntary payments. However, if such unions endure, couples usually engage in other long-term exchanges with affines. Today extremely extravagant bridewealth exchanges are made by the wealthy to pursue political or business goals but they also have high rates of adultery, second wife problems and divorce. The male privilege of polygyny remains, although in practice it is no longer restricted to leaders but rather open to any men with new-found wealth from industry, business or public service.

If norms and values are maintained by third-party punishment in small-scale societies (Boehm, 2012; Boyd et al., 2003; Fehr & Gächter, 2002), why do communities not exert sanctions and stop such infractions? There are two primary reasons. One lies in the level of social regulation for marriage. It is parents and immediate family members who control marriage. Although parents may try to block *de facto* relationships by initial punitive responses or disownment, there is simply too much to lose from rejection after years of investment and caring. Parents wait to see if these unions will last and accept them when children are born. The second reason that third parties are reluctant to become involved in the family problems of others is because sanctioning is expensive, does not promote cooperation and may create rifts to the detriment of their own interests (Wiessner, 2020). In contrast, alterations of norms held by groups – defending clan members right or wrong, cooperation in war and peace making or land matters – occur much more slowly, even though the impact of guns, mobile phones and cash is daunting. People cannot afford to depart from norms that are upheld by groups who are essential to their survival.

What do people seek to preserve as they adapt to changing times? Because families and community can do little through coercion or punishment to enforce compliance to marital institutions, the greatest efforts are made to reduce the impact of infractions on community cooperation and cohesion. Magistrates and community members take the time to participate in the public forums of customary courts, listening to both sides respectfully, hearing the opinions of others, providing evidence and making suggestions (Wiessner, 2020). And they do not stop there. After settlements are made, third parties contribute wealth to the compensation to make up for harm done by domestic violence or adultery, to deter retaliation, reintegrate wrongdoers and reduce the chances that marital conflicts will escalate. Enduring marriages are desired but the big picture – community cooperation and preservation of social ties – is prioritised.

Customary courts hold the reins of peace in Enga; however, what they have not been able to do is to reduce the high rates of domestic violence that have reached epidemic proportions throughout Papua New Guinea (Ganster-Briedler, 2010; Goddard, 2009; Jolly et al., 2012; Lewis et al., 2008; MacIntyre, 2012). Why? The primary factor, particularly in the Papua New Guinea Highlands, is a long tradition of male privilege and dominance (Gibbs, 2016; Zimmer-Tamakoshi, 2012). As Martha MacIntyre (2012, p. 262) has pointed out, for greater gender equality men must relinquish privileges maintained by the threat of violence and change behaviour that has deep cultural roots (Hukula, 2012). Underlying attitudes remain long after cultural institutions have dissolved; fears and biases generated by former male cults linger, hindering adaptation. Socialisation of children involves harsh physical punishment in many Papua New Guinea societies; violent responses are common within and between genders and generations. These cultural factors are accentuated by the role of money in unequally determining male success, women moving into male roles, high degrees of mobility, suspicion, rumours spread by mobile phones, poverty and substance abuse. Customary courts can hand down substantial settlements for domestic violence and repeated offenders may cut be off by community members, but this may not deter actions that are set off by rage, not rationale.

No easy solutions to the current problems of second wives, adultery and domestic violence are in sight. There are some efforts afoot in Papua New Guinea to confront marital problems through instituting new overarching rules. Bans on polygamy and registration of customary marriages have been seriously considered by the government for decades (Kidu, 2000), but there has been little progress owing to opposition and difficulties in implementation. Churches, non-governmental organisations and government are making concerted efforts to address the problem of domestic violence country-wide with recent efforts to change male attitudes. In Enga the many denominations of churches, religious movements and groups from civil society are seeking to restore order to Enga society. Some have had a significant impact on current practices, including preaching conservative sexual mores, forbidding alcohol, smoking and betelnut, and in some cases discouraging compensation. Others have introduced some truly odd ideas that can contribute to AIDS accusations and witchcraft hysteria. Traditional institutions governing marriage are easily dismantled in rapidly changing times, but new ones are not easy to build because of wide cultural differences between past and present ideals regarding patriarchy, gender relations, the roles and obligations of spouses, and sexual mores. Culture matters. The days of the spirit woman who married bachelors and transformed them into handsome men, respectful husbands and loyal clan members have come and gone; it will take an arsenal of modern institutions, government, churches, non-governmental organisations and groups from civil society to replace her.

## Materials and methods

In 2007, data for 402 women were collected by Lelyame Yoane, Wilhelmin Anton, Pesone Munini and Polly Wiessner to obtain approximate age, spouses’ clans and residences, who arranged the marriage, whether bridewealth was paid, whether divorce occurred and why, and number of children born. Between 2008 and 2019, observational data was collected for: (a) 533 court hearings in the Wabag OMS, the provincial capital by Nitze Pupu with assistance from Anton Yongapen and Polly Wiessner and (b) 333 VC cases in three communities in the adjacent Ambum Valley by Larsen Kyalae with assistance from Polly Wiessner between 2008 and 2020. Points recorded include name of complainant and defendant, clans, demographic details and relationship of contesting parties, complaint, request of complainant, response of the defendant, witnesses and evidence presented, agreement, settlement, amount of settlement, reasons for settlement, crowd response, satisfaction and police involvement (Supplementary Material 2). All OMS and VC hearings held on a certain day were observed without prioritising particular complaints. In 2016 we began the time-consuming job of following up settlements to see how much compensation was actually paid, who helped with payments, the relation between the two parties, reasons for helping (or not helping) and satisfaction with the settlement. Texts from case descriptions have been entered into a database in Filemaker Pro and coded by Wiessner. The research was approved by the University of Utah Institutional Review Board #00012979. Because most participants were illiterate or semi-literate, they were consented with discussion of the research and verbal consent on audio-recordings.

## Supplementary material

For supplementary material accompanying this paper visit https://doi.org/10.1017/ehs.2021.13. click here to view supplementary material

## Research transparency and reproducibility

Not reproducible – observational data of court cases.
