# Supplementary material for: When marital institutions break down: Impact and adaptation among the Enga of Papua New Guinea
Source: Evol Hum Sci. 2021 Mar 1;3:e19. doi: 10.1017/ehs.2021.13 (PMC10427295; doi:10.1017/ehs.2021.13)
Supplement: Supplementary file 1 [file S2513843X2100013Xsup001.docx]

**Supplement 1. Photos from customary courts.**

**Photo 1. Village court magistrates returning from settling a tribal conflict.**

**
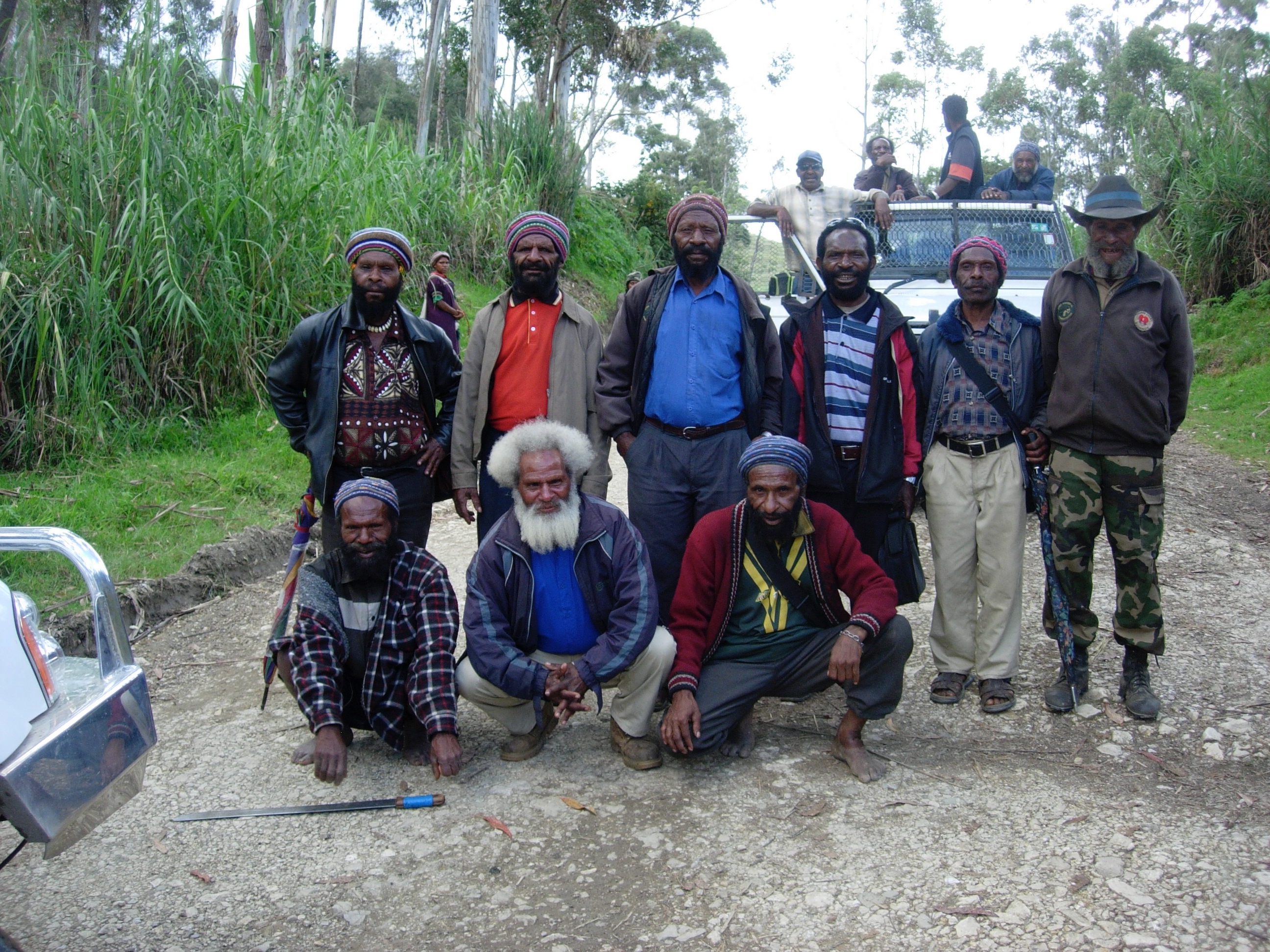
**

**Photo 2.** **Village court magistrates in conference with National and Supreme Court Judge, Justice Regina Sagu to discuss coordinating customary and formal courts on land issues.**

**
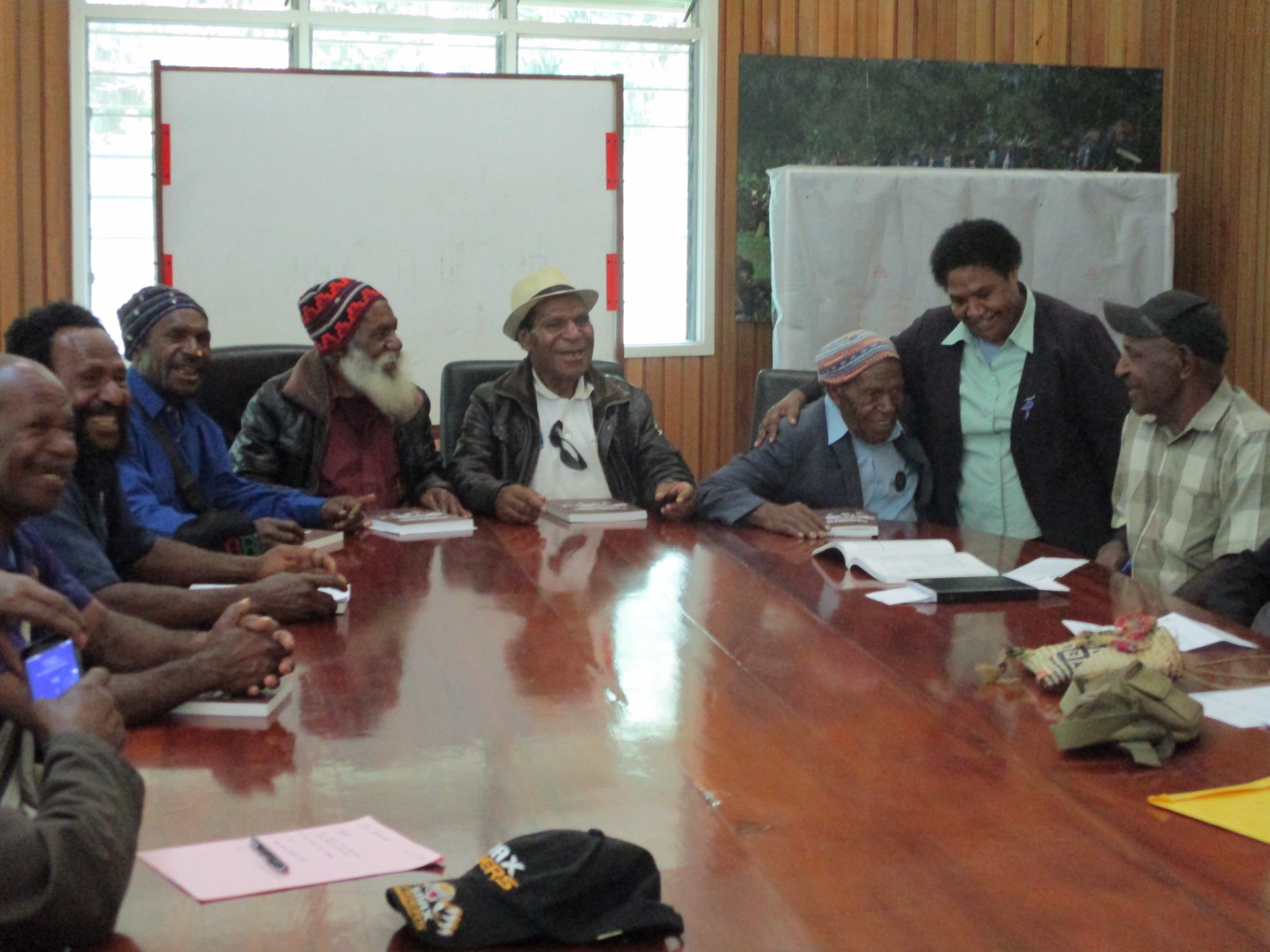
**

**Photo 3. A woman with something to say at a village court hearing.**
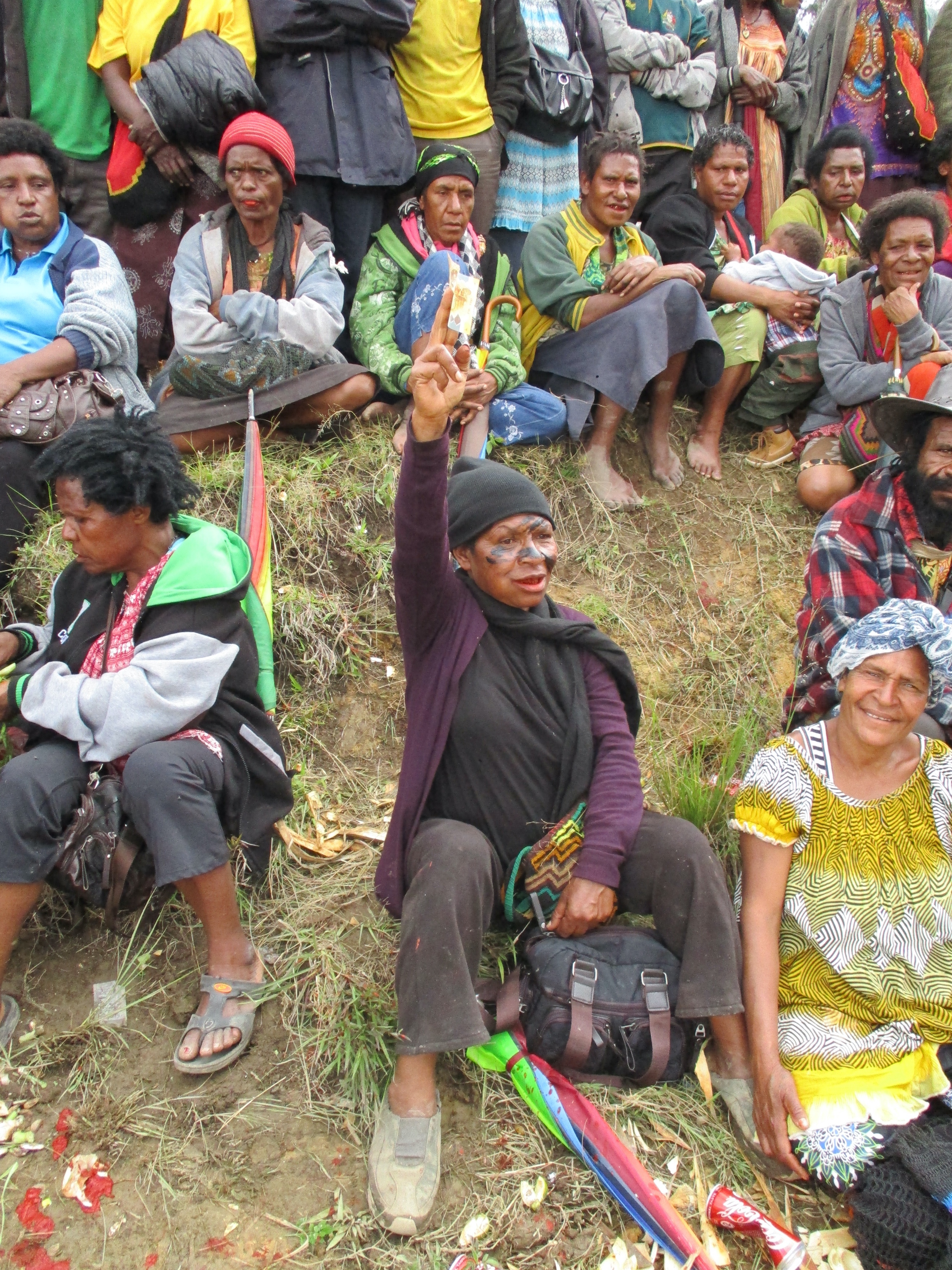


**Photo 4. Contributing to compensation.**


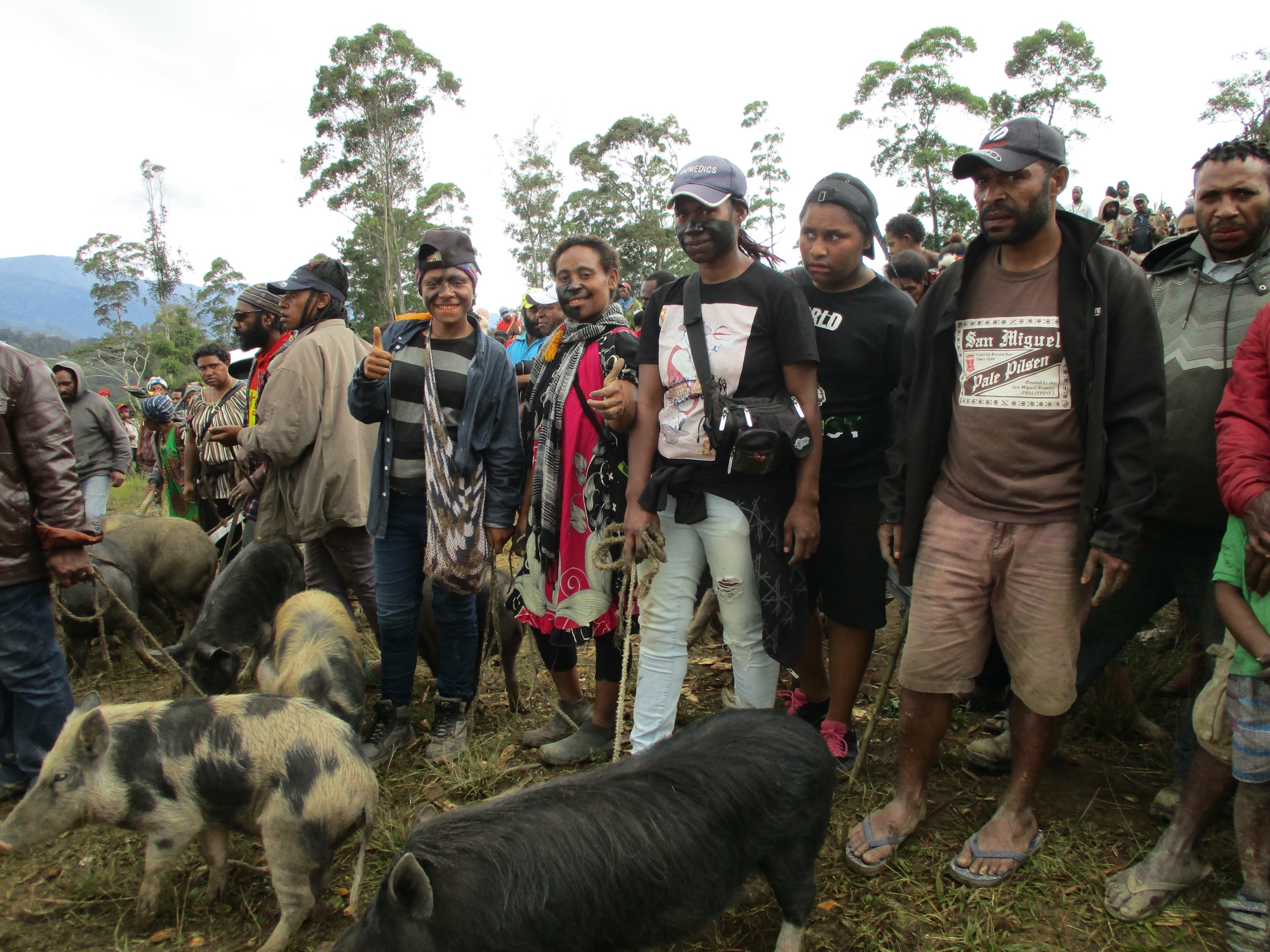


**Photo 5. The inevitable afternoon rain that disrupts village court sessions**.

.
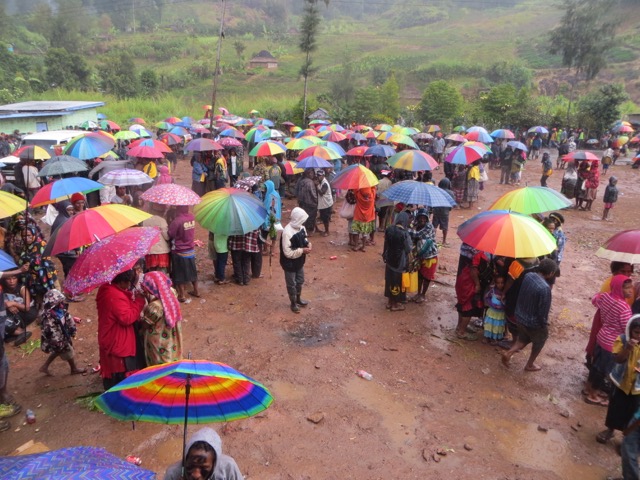


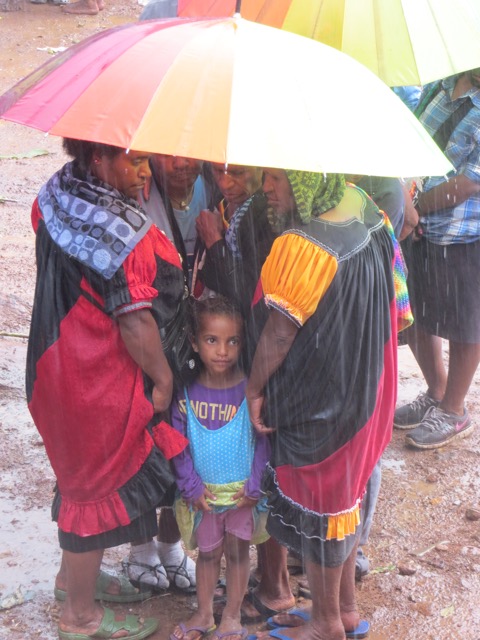


**Supplement 2. Examples of court cases and information recorded.**

**Case1. Conflict between co-wives**

**Complainant:**

Gender: Female

Age: 20-25

Education: unknown

Profession: subsistence farmer

Clan: xxx

**Defendant:**

Gender: female

Age 20-25

Clan:xxx

Education: unknown

Profession: Subsistence Farmer

Relation: co-wives

**Complaint:** Both parties argued after the complainant and first wife gave a plate of rice to her husband that she had cooked for him and the husband gave the plate to the defendant, his second wife, to eat. The next day, the jealous complainant got angry and attacked the defendant. In response, the defendant beat the complainant up. The complainant then mobilized her sisters and husband’s mother, wounding the defendant seriously on her hand and head.

**Request:** Compensation

**Response of defendant**: The defendant refused to pay compensation to the complainant. She asked the Court to order the complainant to pay her compensation for wounding her hand and head with a bush knife. A few members of the crowd said both parties must pay compensation to each other. The husband of both parties came as a witness for the defendant. He said the complainant must pay compensation to the defendant because the defendant nearly lost her life when the complainant and her sisters used a bush knife to chop the defendant.

**Settlement:** The VC ordered both wives to pay one hundred kina each. Additionally, the mother of the husband who participated in the attack and wounded the defendant must pay fifty kina. The Court also gave an order for the complainant to pay five hundred kina to the defendant. The complainant was given bride price and the defendant was not. This helped to prevent the complainant from attacking the defendant seriously; the mother of the husband helped the complainant was the one who wounded the defendant.

Defendant to pay:
100 kina
Complainant to pay:
100 kina + 500 kina
Mother of husband to pay:
50 kina

**Satisfaction:** The complainant was not satisfied and said their husband must pay compensation to her. Complainant said she will pay compensation to defendant.

**Crowd response**: The defendant and complainants relatives in the crowd shouted at each other. The crowd said the husband created the trouble so he must pay compensation to both parties. The crowd said the magistrates came up with the wrong decision, that the husband should also pay. The case was difficult because both parties wanted compensation from each other. A neutral observer (age 50-55) said the complainant and the husband of both wives must pay compensation because he did not pay any bride wealth to the defendant, and in this incident the defendant nearly lost her life. Another neutral observer (age 40-45) thought the case should be taken to OMS in Wabag but first the defendant must go to Kandep and inform her parents before the Court handed a decision down. Young girls are getting married for money these days and they don't care much about their lives and parents.

**Police involvement**: none

**Outcome:** This case was not followed up to track outcome except to note that in such cases it is the responsibility of the husband to settle problems between co-wives.

**Case 2. Adultery and domestic violence**

**Complainant**

Name:

Age: 30 – 35

Sex: Female

Clan: xxx

Education: 0

Profession: Farmer

**Defendant**

Name:

Age: 30 – 35

Sex: Male

Clan: XXX

Education: Grade 6 Drop out

Profession: Self-employed

**Complaint/dispute/offense** (include conditions of offense):

Defendant had a love affair with another woman and beat up his wife (complainant). She had cuts and bruises on her head. The complainant took the matter to court. The other woman who was pregnant and became his second wife.

**What was the complainant asking the court to do?**

Complainant asked the court order the defendant to pay her compensation for the cuts on her head and for beating her up.

**How did the defendant respond?**

was guilty and he agreed to compensate her.

**Did the magistrates ask the crowd? Did anybody respond? What did they say?** Complainant’s father asked the defendant to repay the two pigs which he had given the defendant for the funeral feast of father’s brother.

**Who were the witnesses and what was the evidence?**

No witnesses because guilt was omitted.

**What was the settlement or outcome?**

The court ordered the defendant to pay four (4) pigs and cash amount of K1, 500.00 to compensate the complainant after one month.

**Crowd response?**

Two leaders from the community said that the defendant must pay more pigs and money to the defendant as the injuries were severe and she could have lost her life.

**Were there disturbances?**

No disturbances

**Were both parties satisfied?** Why not?

Both parties were satisfied.

**Was the case difficult and why?**

No, it was not difficult

**Reasons for decision. Values behind decision.**

To prevent the complainant from attacking the defendant’s new wife (the one he had the affair with) who had a new baby and to save the marriage.

**Length of time.**

Two hours

**Notes:** Such conflict often occurs when men have affairs and say they are marrying second wives. Sometimes domestic violence occurs to try to drive away the first wife. The compensation is for adultery and marrying a second wife without permission of the first.

**Outcome:**

Five hundred and eighty kina more that the order were paid were paid as the crowd suggested. Five major contributors helped the defendant. He was from a distant clan and lived in his wife’s place. Reasons given for contributing were that he had been generous in helping others in the past, to show leadership in solving community problems and to show sympathy for the complainant and promote peace and cooperation in the community.

**Case 3. Drunkeness and domestic violence**

**Complainant**

Name:

Age: 25-30

Sex: Female

Clan: xxx

Education: 12

Profession: Small business

**Defendant**

Name:

Age: 30 – 35

Sex: Male

Clan: XXX

Education: Higher Education

Profession: student in technical college

**Complaint/dispute/offense** (include conditions of offense):

The defendant came home drunk and beat up his wife who was 4 months pregnant.

**What was the complainant asking the court to do?**

Pay compensation for domestic violence a and to stop drinking alcohol.

**How did the defendant respond?**

He admitted his guilt and apologized

**Did the magistrates ask the crowd? Did anybody respond? What did they say?**

The defendant felt guilty and promised to pay compensation. The clansmen and two magistrates gave the orders to the defendant to pay compensation. The father of the defendant came as a witness of the complainant and said the defendant got drunk and beat the complainant. He said he witnessed the incident himself and the defendant must pay the complainant compensation.

**Who were the witnesses and what was the evidence?**

Defendant’s father was the witness and the defendant admitted his guilt.

**What was the settlement or outcome?**

Court ordered the defendant to pay five hundred kina. One week later, the defendant paid one pig worth two thousand kina plus eleven hundred kina in cash to the complainant.

**Crowd response?**

The defendant's father does not want the complainant to divorce the defendant because she usually uses her money to pay school fees for the defendant, and built a house for both parties to live and buys food for all of the family to eat. Also other in the crowd said that it was a good marriage that provided useful social ties.

**Were there disturbances?**

No disturbances

**Were both parties satisfied?** Why not?

Both parties were satisfied as was the crowd. The crowd said the Court came up with a good settlement using the complainant's witnesses. People contribute to the compensation

**Was the case difficult and why?**

No, it was not difficult

**Reasons for decision. Values behind decision.**

Domestic violence during pregnancy was intolerable and to be well compensated.

**Length of time.**

Two hours

**Notes:** The defendant is a good community member and husband but can be violence when drunk.

**Outcome:**

PGK2600 more than the court order were paid with three major contributors and more smaller ones. Reasons for contribution were to protect an otherwise good marriage that provided important ties for the community. Even the family of the defendant was outraged by the incident though the wife was not severely injured.

**Case 4. Marital dispute and violence between repeated troublemakers**

Date: xxx

Complainant:

Age 25-30

Clan: xxx

Profession : self-employed, subsistence farmer

Education: Grade 10

Relationship: Married couple

**Defendant:**

Gender: male

Age: 30-35

Clan:xxx

Education: grade 10

Profession: carpenter

**Complaint:** The defendant did not give money to his wife for some time, so she got frustrated and swore in public using defamatory words like- big penis, dog-man, and HIV/AIDS carrier.  The defendant was so ashamed in public that he chopped the complainant's head with a bush knife.  She was hospitalized and upon discharge brought the matter to the VC.

**Request:** Compensation for domestic violence in response to slander. Complainant asking defendant to compensate her for using an offensive weapon in public and cutting her body.

**Defendant’s response**: The defendant also asked the Court to order the complainant not to use abusive language in public.

**Evidence:** Happened in public. The younger sister of the complainant also stated the defendant refused to give the complainant money when she asked him to buy vegetables at the market.  In return, she swore at him.

**Crowd response**: Four people from the crowd said the cause of the problem was because the defendant failed to give the complainant money.  As a result, she swore in public.  Therefore, to solve the problem, the defendant must give the complainant some money.  The younger sister of the complainant stated the defendant refused to give the complainant money when she asked him to buy vegetables at the market.  In return, she swore at him.

**Settlement:** The Court ordered the defendant to pay five hundred kina in cash and one pig worth four hundred kina.  Additionally, the complainant was ordered not to use abusive language again.

**Reason**: To prevent the complainant from divorcing the defendant.

**Police:** none

**Satisfaction**: Both parties were satisfied. A few people from the crowd seemed happy with the court order.

**Follow up**. In the follow up we found that K300 less was paid than ordered. The reason was that this couple was always getting into trouble, did not give generously to others, and so nobody came to assist them.
